# Perspectives of pediatric patients with inborn errors of metabolism on long-term treatment and metabolic emergency management

**DOI:** 10.1186/s13023-025-04046-y

**Published:** 2025-10-06

**Authors:** Tanjana Harings, Thilo Bertsche, Alena Gerlinde Thiele, Wieland Kiess, Astrid Bertsche, Skadi Beblo, Martina Patrizia Neininger

**Affiliations:** 1 Drug Safety Center, Leipzig University and Leipzig University Hospital, and Clinical Pharmacy, Institute of Pharmacy, Medical Faculty, Leipzig University, Bruederstrasse 32, 04103 Leipzig, Germany; 2 Center for Pediatric Research, University Hospital for Children and Adolescents, Leipzig University Hospital, Liebigstrasse 20a, 04103 Leipzig, Germany; 3 Division of Neuropediatrics, University Hospital for Children and Adolescents, University Medicine Greifswald, Ferdinand-Sauerbruch-Strasse 1, 17475 Greifswald, Germany; 4 Center for Rare Diseases, Leipzig University Medical Center, Philipp-Rosenthal-Strasse 55, 04103 Leipzig, Germany

**Keywords:** Inborn errors of metabolism, Pediatrics, Long-term treatment, Medication, Metabolic emergencies, Patient education

## Abstract

**Background:**

Long-term treatment and emergency management are essential in most pediatric patients with inborn errors of metabolism (IEM). In routine care, these patients receive age-appropriate education to support adherence and self-management. However, little is known about pediatric patients’ perspectives on long-term treatment and emergency management. We explored patients’ perspectives to identify individual needs and challenges.

**Methods:**

After ethical approval and written informed consent, we conducted semi-structured interviews with 79 patients diagnosed with IEM, aged ≥ 6 years. The interview consisted of two parts: long-term treatment (Part A) and emergency management (Part B).

**Results:**

Altogether, 79 patients participated. Part A on long-term treatment was completed by 66 patients. Of those, 70% (46/66) reported regular medication intake. While 67% (44/66) experienced their medication as beneficial, 24% (16/66) were unsure whether their medication was helpful. However, adhering to their prescribed treatment regimen was very important for 74% (49/66), mainly to prevent adverse health outcomes (48%, 32/66). Nearly half of patients (48%, 32/66) reported experiencing burden related to their medication, primarily due to its constant presence in everyday life (26%, 17/66) and unpleasant taste (13%, 9/66). Among 42 patients at risk of metabolic emergencies, 62% (26/42) were aware of the risk and were asked about their emergency management in Part B of the interview. Of those, 54% (14/26) were able to describe symptoms and their actions in response. Overall, patients rated their level of preparedness for emergencies as “moderate”.

**Conclusion:**

Pediatric patients with IEM demonstrated a good understanding of and positive attitude toward their medication. However, despite education as part of routine care, gaps remain in awareness and preparedness regarding metabolic emergencies that require further support for the affected patients.

**Supplementary Information:**

The online version contains supplementary material available at 10.1186/s13023-025-04046-y.

## Introduction

Inborn errors of metabolism (IEM) constitute a diverse group of rare genetic disorders that impair metabolic pathways, resulting in the accumulation of toxic compounds or a deficiency of essential metabolites. The treatment of IEM aims to restore metabolic balance and often includes dietary restrictions and lifelong medication as part of long-term treatment [1–3]. Consequently, patients with IEM are faced with demanding treatment regimens that can significantly impact daily life [2, 4].

Adherence to treatment can be difficult, particularly during adolescence [2, 4, 5]. It is influenced by various factors, including the patient’s understanding of the condition and medication, experience of therapeutic benefits or adverse drug reactions (ADR), and parental beliefs about treatment [6–8]. Parents generally express positive attitudes toward their children's medication, valuing its effectiveness and importance; however, concerns about ADR and complications with medication intake are common [9]. However, little is known about how pediatric patients themselves perceive their long-term treatment.

Some IEM are associated with the risk of acute metabolic emergencies due to biochemical imbalance during catabolic states (e.g. intercurrent infections). These situations can be life-threatening and may occur even with strict adherence to treatment [10, 11]. Therefore, it is imperative to prevent such emergencies to the extent possible, and to promptly recognize and treat them when they arise [11]. Previous research has shown that parents are generally well informed and confident in recognizing and managing metabolic emergencies [12]. However, there is little evidence on how pediatric patients perceive and manage metabolic emergencies.

Studies have highlighted the disparity between the perspectives of chronically ill children and their parents, encompassing both rare diseases and more prevalent conditions such as epilepsy [13–17]. These discrepancies underscore the importance of directly assessing the pediatric perspective.

To ensure appropriate long-term outcomes, enhance adherence and patient-centered care, as well as foster independence to facilitate the transition to adulthood, it is important to comprehend pediatric patients’ perspectives. This study therefore aimed to explore pediatric patients’ perspectives on long-term treatment and emergency management through semi-structured interviews, with the goal of identifying individual needs and potential challenges.

## Methods

### Patients and setting

After approval by the local ethics committee, all patients scheduled for an appointment at a university hospital’s pediatric outpatient clinic for metabolic disorders between June 15, 2022 and June 14, 2023 were consecutively invited to participate in our study. To be eligible, patients had to have a diagnosis of an IEM and fulfil at least one of the following criteria: use of long-term medication (including medicinal products, vitamins, mineral supplements, or amino acids), or an IEM associated with metabolic emergencies. Further inclusion criteria were age ≥ 6 years, school attendance, and sufficient cognitive ability to understand and answer interview questions. Patients who had multiple appointments during the study period were included only once. Written informed consent was obtained from all participants and their parents.

As part of routine care at the outpatient clinic, pediatric patients received regular, age-appropriate education on long-term treatment and emergency management, beginning no later than school enrolment.

### Semi-structured interview

We conducted semi-structured interviews based on a predefined questionnaire (Supplementary file 1). The interview included two parts:

Part A: Long-term treatment (conducted only in patients taking at least one medication as long-term treatment according to medical record and self-report).

1. Awareness and understanding of long-term treatment
2. Perceptions of long-term treatment and daily routine
3. Experienced burdens with long-term treatment
4. Experienced and feared ADR

Part B: Emergency management (conducted only in patients with IEM associated with metabolic emergencies, according to diagnosis and self-report).

1. Awareness of metabolic emergencies
2. Perception and management of metabolic emergencies

The interview was based on a previous study conducted among parents of pediatric patients with IEM [9] and was adapted to ensure age appropriateness for the entire range from 6 to 17 years. It was revised following expert panel evaluation during pretesting. The interview consisted primarily of open-ended questions that allowed multiple responses to capture the quality and range of patients’ responses. The aim was to assess patients’ subjective perceptions rather than medical accuracy. Responses to open-ended questions were categorized thematically by two independent raters. Discrepancies were discussed until consensus was reached.

Patients used visual aids to respond to Likert scale questions (0 = not at all to 5 = very well/very regularly/very much/very important/always, depending on context). Parents were asked not to interfere and completed a separate questionnaire on the child’s sociodemographic data concurrently. Additional data (diagnosis, prescribed medication, sociodemographic data) were collected from the patients’ medical records.

### Analysis and statistics

Calculations were performed using SPSS (Statistical Package for the Social Science, Version 29.0.1.0, IBM, Armonk, New York, USA) and Microsoft Excel 2019 (Microsoft Corporation, Redmond, Washington, USA). Frequencies are shown as numbers and percentages. Continuous data are presented as median with first and third quartiles (Q25/Q75) and minimum/maximum (min/max). Group comparisons by age (6–11 vs. 12–17 years), gender (male vs. female), and previous experience with metabolic emergencies (yes vs. no) were conducted using Mann–Whitney U tests. A p-value ≤ 0.05 was considered to indicate significance.

## Results

### Characteristics

**Fig. 1:**
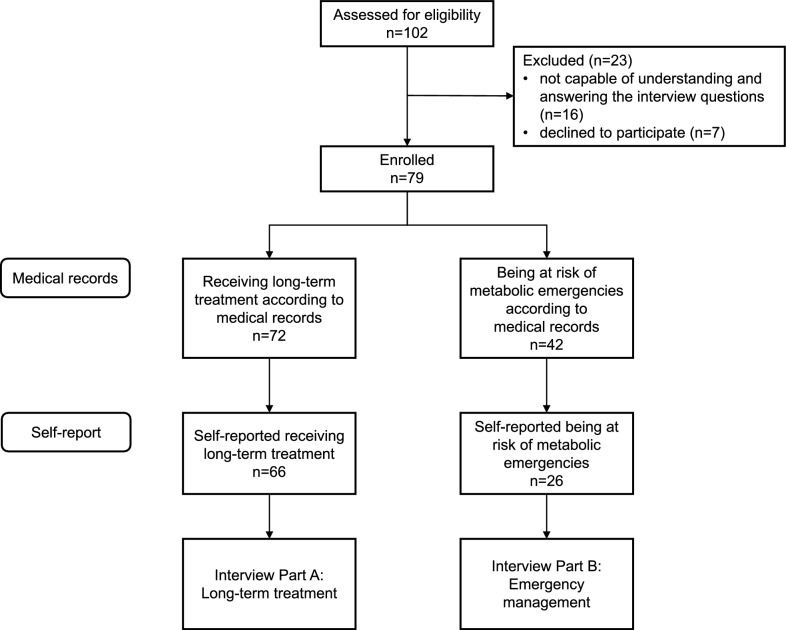
Study inclusion and allocation flowchart

Of the 86 eligible patients who visited the hospital during the study period, 79/86 (92%) agreed to participate in the study and were enrolled accordingly. Part A of the interview was completed by 66/79 (84%) patients and Part B by 26/79 (33%) patients (Fig. 1). Patient characteristics are shown in Table 1.

**Table 1:** Patients’ characteristics

Characteristics	Patients Part A: long-term treatment	Patients Part B: emergency management
Total number of patients [n]	66	26
Median age (Q25/Q75; min/max) [years]	12 (10/14; 7/17)	11 (9/14; 7/16)
Gender [n (%)]		
Female	32 (48)	13 (50)
Male	34 (52)	13 (50)
Education [n (%)]		
Primary school	17 (26)	9 (35)
Middle school	22 (33)	12 (46)
Grammar school	16 (24)	4 (15)
Vocational school	4 (6)	0 (0)
Special needs school	7 (11)	1 (4)
Diagnosis [n (%)]		
3-MCC deficiency	2 (3)	1 (4)
ASA deficiency	1 (2)	0 (0)
BD	3 (5)	0 (0)
CUD	2 (3)	0 (0)
CUD and Severe neonatal vitamin B12 deficiency	1 (2)	0 (0)
FHI	0 (0)	1 (4)
FH	8 (12)	0 (0)
Galactosemia	1 (2)	0 (0)
GA1	1 (2)	1 (4)
GSD1a	2 (3)	2 (8)
GSD1b	1 (2)	1 (4)
GSD type IX	0 (0)	1 (4)
IVA	1 (2)	1 (4)
Kabuki syndrome	1 (2)	0 (0)
LCHADD	2 (3)	1 (4)
LAL deficiency	1 (2)	0 (0)
MCADD	11 (17)	13 (50)
MCADD and SLOS	1 (2)	1 (4)
MERRF	1 (2)	0 (0)
MODY	1 (2)	0 (0)
PKU	19 (29)	0 (0)
Tyrosinemia type I	2 (3)	0 (0)
Vitamin B12 and folic acid deficiency	1 (2)	0 (0)
VLCADD	2 (3)	3 (12)
Unexplained hypoglycemia	1 (2)	0 (0)

*3-MCC deficiency* 3-Methylcrotonyl-CoA-carboxylase deficiency, *ASA deficiency* Arginino succinic acid deficiency, *BD* Biotinidase deficiency, *CUD* Carnitine uptake deficiency, *FH* Familial hypercholesterolemia, *FHI* Familial hyperinsulinemic hypoglycemia, *GA1* Glutaraciduria type I, *GSD1a* Glycogenosis type Ia, *GSD1b* Glycogenosis type Ib, *GSD type IX* Glycogenosis type IX, *IVA* Isovaleric academia, *LAL deficiency* Lysosomal acid lipase deficiency, *LCHADD* Long chain 3-hydroxyacyl-CoA dehydrogenase deficiency, *MCADD* Medium-chain-acyl-CoA-dehydrogenase-deficiency, *MERRF* Myoclonus epilepsy associated with ragged-red fibres, *MODY* Maturity-onset diabetes of the young, *PKU* Phenylketonuria, *SLOS* Smith–Lemli–Opitz syndrome, *VLCADD* Very-long-chain-acyl-CoA-dehydrogenase deficiency

### Part A.1 Awareness and understanding of long-term treatment

According to the medical records, 72/79 (91%) patients were receiving long-term treatment for their IEM. Of these, 66/72 (92%) confirmed this in the interview. Among them, 53/66 (80%) were able to name their medications precisely, while 6/66 (9%) provided a general description of their medication, e.g. “tablets,” “vitamins” or “hormones,” In response to the question, “Why should you take your medication regularly?”, 45/66 (68%) patients explained their point of view. These explanations were categorized and are summarized in Table 2.

**Table 2:** Patients’ explanations for the importance of regular medication intake

Category	Examples of explanations	Patients (N = 66), n (%)
Substitution	• “So that I still get the supplements that I am not allowed to eat.” • “So that I get the substitute for protein.” • “That my body metabolizes the proteins.” • “Because biotin is not produced in my body.” • “I have too little something in my body, (my medicine) replaces that.”	18 (27)
Improvement or normalization of the condition	• “So that my levels are not so high, and I can eat protein.” • “So that I can eat normally.” • “So that I don’t have to get up at night to eat something.”	17 (26)
Prevention of deterioration	• “So that I don’t have to go to the hospital.” • “So that I don’t get sick.”	8 (12)
Other	• “Because of my disease.” • “That I grow, and everything is good in my body.”	8 (13)
“I don’t know”		22 (33)

Responses were categorized thematically. Multiple categories per patient were possible

### Part A.2 Perceptions of long-term treatment and daily routine

**Fig. 2:**
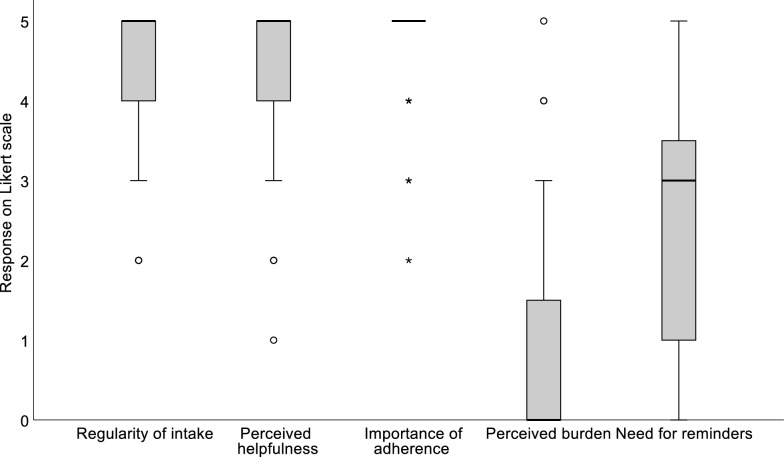
Patients’ perceptions of various aspects of their long-term treatment. Responses were given on a Likert scale ranging from 0 = not at all to 5 = very well/very regularly/very much/very important/always, depending on question context

Figure 2 summarizes patient-reported experiences and attitudes regarding long-term treatment and daily routine. Most patients reported taking their medication “very regularly” (46/66, 70%) and rated adherence as “very important” (49/66, 74%). The primary reasons provided by the patients for the importance of adherence included preventing adverse health outcomes (32/66, 48%), improving or normalizing their IEM (9/66, 14%) or, “because my physician told me to do so” (9/66, 14%). Of the patients, 44/66 (67%) stated that their medication helped them “(very) well,” while 16/66 (24%) responded, “I don’t know,” when asked about the helpfulness of their medication. Regarding reminders, 32/66 (48%) of patients reported needing reminders rarely or never, whereas 16/66 (24%) indicated needing reminders (almost) always. No statistically significant differences were found between age groups or gender in responses related to treatment perceptions and daily routine.

### Part A.3 Experienced burden with long-term treatment

While 34/66 (52%) patients reported that medication intake did not bother them at all, 32/66 (48%) described experiencing a burden associated with their long-term treatment (Fig. 2). The most frequently mentioned aspects were the constant presence of medication in everyday life (17/66, 26%) and its unpleasant taste (9/66, 14%; Table 3).

**Table 3:** Reported burden associated with medication intake

Category of perceived burden	Example statements	Patients (N = 66), n (%)
Constant presence in everyday life	• “That I have to take it every day again.” • “That I always have to remember.” • “Taking this every day is annoying.”	17 (26)
Unpleasant taste	• “The taste.” • “Because the medicine tastes bitter, I don’t like it.”	9 (14)
Social aspects	• “Other children do not have to take it.” • “At school, when the others see it.”	4 (6)
Difficulties with swallowing	• “Sometimes I can’t swallow the tablets.” • “Swallowing tablets with braces is difficult.”	3 (5)
Sleep disruption	• “That I also have to take them at night.”	2 (3)
Forgetting	• “Because sometimes I forget to take it and then I have to take it early in the morning.”	2 (3)
Other	• “It sticks and you have to scrub the cup properly.”	3 (5)
“I don’t know”	Not applicable	1 (2)

Responses were categorized thematically. Multiple categories per patient were possible

### Part A.4 Experienced and feared ADR

Of the patients, 4/66 (6%) reported fears of ADR, with 3/66 (5%) mentioning non-specific ADR. Of the patients, 10/66 (15%) had already experienced ADR associated with their IEM medication, including: abdominal pain (4/66, 6%), nausea (2/66, 3%), and tiredness, fish odor, flushing and “rough patches on the ankle” (1/66, 1% each).

### Part B.1 Awareness of metabolic emergencies

According to medical records, 42/79 (53%) patients were at risk of metabolic emergencies due to their IEM. Among these, 26/42 (62%) patients were aware of their risk. The remaining patients either assumed their IEM was not associated with metabolic emergencies (8/42, 19%) or were unsure (8/42, 19%). No statistically significant differences were found between age group, gender or a previous experience of a metabolic emergency in the patients’ responses regarding awareness.

### Part B.2 Perception and management of metabolic emergencies

Among the patients who were aware that their IEM was associated with metabolic emergencies, 11/26 (42%) reported having already experienced such an event. When asked how they would recognize an occurring metabolic emergency, 14/26 (54%) patients were able to describe possible symptoms and what actions they would take in response to them (Table 4). The most frequently mentioned responses included eating something (5/14, 36%), telling someone (5/14, 36%) or taking medication (4/14, 29%). According to medical records, 26/26 (100%) patients had been provided with an emergency plan. However, when asked about the existence of such a plan, 9/26 (35%) patients confirmed having one. Patients felt “moderately” prepared to respond to a metabolic emergency (6-point Likert scale; median 3; Q25/Q75 2/4; min/max: 0/5). Of the patients, 5/26 (19%) were unable to assess their level of preparedness. No statistically significant differences were found between age group, gender or a previous experience with a metabolic emergency in the patients’ responses regarding recognition, response, or preparedness concerning metabolic emergencies.

**Table 4:** Patients’ descriptions of symptoms indicating a metabolic emergency and their response actions

Category of symptoms	Patients (N = 14), n (%)
Nausea	4 (29)
Tiredness	4 (29)
Trembling	3 (21)
Weakness	3 (21)
Abdominal pain	1 (7)
Palpitations	1 (7)
Feeling unwell	2 (14)
Headache	2 (14)
Hunger	2 (14)
Impairment of attention	1 (7)
Muscle pain	1 (7)
Restlessness	1 (7)
“Stroke”	1 (7)

Category of actions in response	Patients (N = 14), n (%)
Eat something	5 (36)
Tell someone	5 (36)
Take medication	4 (29)
Take a rest	3 (21)
I don’t know	1 (7)

Responses were categorized thematically. Multiple categories per patient were possible

## Discussion

The findings of this study offer a comprehensive understanding of the perspectives of pediatric patients with IEM. Most patients understood the necessity of their medication and reported adherence to their physician’s instructions, often driven by the desire to prevent adverse health consequences. About half of the patients felt bothered by the medication’s constant presence in everyday life and unpleasant taste. Two-thirds of the affected patients were aware of their risk of metabolic emergencies, but only half of those aware of the risk could also describe symptoms and how they would respond to metabolic emergencies.

### Long-term treatment

For patients with IEM, adherence to long-term treatment is essential to ensure good development and survival [2]. Important factors for promoting adherence in pediatric patients with chronic diseases include knowledge about the disease and medication, and a positive attitude toward the medication [6–8]. In our study, most patients showed extensive knowledge about their long-term treatment. Two-thirds were able to explain why they needed to take it. Of the patients, 70% stated taking their medication regularly, and approximately half reported they were self-reliant, requiring little or no reminding. For more than 70% of patients, adherence to the prescribed regimen was considered important, primarily to avoid adverse health outcomes. Interestingly, about one-quarter of patients responded, “I don’t know,” when asked whether their medication was helping them. This uncertainty may reflect the nature of many IEM treatments, whose effects are often not immediately observable. Nonetheless, two-thirds of patients reported perceiving their medication as beneficial, suggesting that even without tangible effects they understood its intended purpose and preventive function.

A low frequency of treatment-related difficulties, such as ADR or intake-related burden, has been described as a factor supporting a positive attitude toward medication in pediatric patients [8, 18]. Only a few patients in our study expressed concerns about ADR or reported experiencing them. While half of the patients reported no burden associated with medication intake, the remaining patients felt particularly bothered by its constant presence in everyday life or unpleasant taste.

In summary, pediatric patients demonstrated a good understanding of their long-term treatment and expressed generally positive attitudes. Despite occasional uncertainty about treatment benefits or experiencing burden with medication intake, most participants reported to adhere to medical advice, motivated by awareness of potential health consequences.

### Metabolic emergencies

Metabolic emergencies pose a potentially life-threatening risk to patients and should be prevented whenever possible [11, 19, 20]. If unavoidable, it is essential to recognize and treat them promptly to prevent long-term complications [11]. These emergencies often lead to rapid deterioration, which makes it difficult or impossible for the patients affected to act independently. Consequently, patients with IEM typically receive emergency plans from their physicians, which include both caregiver instructions and guidance for medical professionals.

Parents of patients with IEM demonstrated excellent knowledge about management of metabolic emergencies [12]. However, the patients in our study were attending school, which meant they were not under parental supervision for an extended period each day. Age-appropriate knowledge and awareness of emergency risks are thus essential. Therefore, education on emergency management is provided as part of routine care starting from school age. Despite this routine education, our findings revealed gaps in patients’ awareness, recognition, and response regarding metabolic emergencies. While two-thirds of affected patients were aware of their risk, one-third either lacked awareness or were unsure. Only half of the patients aware were able to describe symptoms indicating a metabolic emergency and explain how they would respond appropriately. Furthermore, although 100% had been provided with an emergency plan, only one-third were aware of its existence. Overall, patients rated their level of preparedness for responding to metabolic emergencies as moderate.

One possible reason for the observed gaps in knowledge and awareness may lie in the structure of early care, which primarily targets caregivers. As patients mature, many express a desire to be more actively involved in medical consultations and decisions regarding their care [21–23]. However, this shift presents a challenge for healthcare professionals: not only must they accommodate the evolving need for autonomy among pediatric patients, but they also have to meet the often divergent information needs of patients and caregivers [23]. Addressing both perspectives within a single consultation requires tailored communication strategies and flexibility in the delivery of medical information. Developing and implementing effective, age-adapted education strategies is therefore essential [23]. Such strategies can help improve pediatric patients’ ability to recognize and respond to metabolic emergencies and strengthen their confidence and preparedness in managing these potentially life-threatening situations.

### Strengths and limitations

A major strength of this study is the inclusion of a diagnostically diverse patient population, allowing for the representation of patients with extremely rare IEM and thereby capturing a broad spectrum of experiences. Future studies could complement this diversity with qualitative interviews in diagnostically homogeneous patient groups to gain deeper, diagnosis-specific insights. However, it is important to acknowledge that some IEM were only represented by single participants, and the study was conducted at a single university hospital. Therefore, the generalizability of the findings may be limited. In addition, long-term treatment in most IEM comprises not only medication intake but also a strict dietary regimen, which is frequently described as a substantial restriction in daily life [2, 4]. Since our study focused on medication, the perspectives reported here may not be directly transferable to patients managed solely through dietary treatment. Furthermore, it cannot be ruled out that patients’ responses may have been influenced by social desirability or other potential biases, such as age, developmental stage, or cognitive abilities.

## Conclusion

Pediatric patients with IEM generally demonstrated a good understanding of and positive attitude toward their medication, despite occasional burden or uncertainty regarding its effects. They were aware of the importance of adherence, but showed limited knowledge and awareness concerning metabolic emergencies, which may reduce their confidence in responding to those situations. To improve long-term health outcomes, support patient autonomy, and facilitate the transition to adult care, effective strategies for age-appropriate patient education should be developed and systematically evaluated.

## Supplementary Information

Supplementary Material 1.

## Data Availability

The dataset generated and analyzed during the current study are not publicly available due to ethical and privacy considerations to protect the confidentiality of participants but are available from the corresponding author on reasonable request.

## Abbreviations

ADR: Adverse drug reaction
IEM: Inborn error of metabolism
