# Supplementary material for: Perspectives of pediatric patients with inborn errors of metabolism on long-term treatment and metabolic emergency management
Source: Orphanet J Rare Dis. 2025 Oct 6;20:502. doi: 10.1186/s13023-025-04046-y (PMC12498459; doi:10.1186/s13023-025-04046-y)
Supplement: Supplementary file 1 — Supplementary Material 1. [file 13023_2025_4046_MOESM1_ESM.pdf]

Additional File 1: Questionnaire. The original questionnaire was conducted in German. We provide a translation to facilitate comprehensibility.

Part A – Long-term treatment (Medication)

1. Do you take medication or supplements for your condition (e.g., tablets, powder, or liquids)?

   Yes / No / Not sure

2. What are the names of your medications or supplements?
3. Why should you take your medication regularly?
4. On a scale ranging from 0 to 5, how regularly do you take your medication or supplements?

   0 = not at all / 1 = very irregularly / 2 = rather irregularly / 3 = somewhat regularly / 4 = rather regularly / 5 = very regularly / Not sure

5. On a scale ranging from 0 to 5, how much do your medications help you?

   0 = not at all / 1 = very little / 2 = rather little / 3 = somewhat / 4 = rather much / 5 = very much / Not sure

6. On a scale ranging from 0 to 5, how important is it to you to take your medication as prescribed by your doctor?

   0 = not at all important / 1 = very unimportant / 2 = rather unimportant / 3 = somewhat important / 4 = rather important / 5 = very important / Not sure

7. Why is it important (or not important) to you?
8. On a scale ranging from 0 to 5, how much does it bother you that you have to take medication?

   0 = not at all / 1 = very little / 2 = rather little / 3 = somewhat / 4 = rather much / 5 = very much / Not sure

9. [If more than 0] What bothers you about taking your medication?
10. On a scale ranging from 0 to 5, how often do you need to be reminded to take your medication?

    0 = never / 1 = very rarely / 2 = rather rarely / 3 = sometimes / 4 = rather often / 5 = always / Not sure

11. Have you ever experienced adverse drug reactions from your medication?

    Yes / No / Not sure

12. [If yes] What kind of adverse drug reactions?
13. Are you afraid of any adverse drug reactions?

    Yes / No / Not sure

14. [If yes] Which ones?

Part B – Emergency Management

1. Can metabolic emergencies occur due to your condition?

   Yes / No / Not sure

2. On a scale ranging from 0 to 5, how well do you feel prepared for such an emergency?

   0 = not at all / 1 = very poorly / 2 = rather poorly / 3 = moderately / 4 = rather well / 5 = very well / Not sure

3. Do you know how to recognize a metabolic emergency?

   Answer options: Yes / No / Not sure

4. [If yes] Please describe the symptoms.
5. What would you do if you noticed those symptoms?
6. Have you already experienced a metabolic emergency?

   Yes / No / Not sure

7. Do you have an emergency plan?

   Yes / No / Not sure
